# Validation of a Spanish translation of the Gratitude Questionnaire (GQ-6) with a Chilean sample of adults and high schoolers

**DOI:** 10.1186/s12955-016-0450-6

**Published:** 2016-03-31

**Authors:** Álvaro I. Langer, Valentina G. Ulloa, José M. Aguilar-Parra, Claudio Araya-Véliz, Gonzalo Brito

**Affiliations:** Laboratory of Experimental Psychology (UACh/CISNe), Faculty of Medicine, Austral University of Chile, Campus Isla Teja s/n, Valdivia, Chile; Red de Salud Mental RedGesam, Santiago, Chile; University of Manchester, Oxford Road, Manchester, M13 9PL UK; University of Almeria, Ctra. Sacramento, s/n, 04120. La Cañada de San Urbano, Almería, Spain; Universidad Adolfo Ibáñez, Escuela de Psicología, Avenida Diagonal Las Torres 2640, Peñalolén, Santiago Chile; Counselling psychologist in private practice, Granada, Spain

**Keywords:** Positive psychology, Gratitude, Adolescence, Validity, Confirmatory factor analysis

## Abstract

**Background:**

Recent studies have associated positive emotions with several variables such as learning, coping strategies or assertive behaviour. The concept of gratitude has been specifically defined as a tendency to recognise and respond to people or situations with grateful emotion. Unfortunately in Latin America, no validated measures of gratitude on different populations are available. The aim of this study was to analyse the psychometric properties of the Gratitude Questionnaire (GQ-6) in two Chilean samples.

**Methods:**

Two studies were conducted: the first with 668 high school adolescents (390 women and 278 men, with ages ranging between 12 and 20, and a mean age 15.54 ± 1.22) and the second with 331 adults (231 women and 100 men, with an average age of 37.59 ± 12.6). An analysis of the psychometric properties of the GQ-6 scale to determine the validity and reliability of the instrument in Chilean adolescents and adults was performed. Bivariate correlations, multiple regression analyses, exploratory factor analysis (EFA) and Monte Carlo simulations were carried out. Finally, a confirmatory factor analysis (CFA) was performed.

**Results:**

A single-factor solution was found in both studies, a 5 item version for the adolescents and 6 items for adults. This factorial solution was invariant across genders. Reliability of the GQ was adequate in both samples (using Cronbach’s alpha coefficient). In addition convergent and discriminate validity were assessed. Additionally, a negative correlation between the GQ-5 and depression in adolescents and a positive correlation between the GQ-6 and happiness in adults was found.

**Conclusions:**

The GQ is a suitable measure for evaluating a person’s disposition toward gratitude in Chilean adolescents and adults. This instrument may contribute to the advancement of the study of positive emotions in Latin America.

**Electronic supplementary material:**

The online version of this article (doi:10.1186/s12955-016-0450-6) contains supplementary material, which is available to authorized users.

## Background

Research on gratitude has flourished in recent years. Gratitude has been defined as ‘a generalized tendency to recognize and respond with gratefulness to the role of other people’s benevolence in the positive experiences and outcomes that one obtains’ [1].

There is a growing body of empirical evidence about the relationship between gratitude and other health-related variables. In a recent study [2] a negative relationship was found between gratitude and depressive symptoms, and this relationship was mediated by other positive emotions and the tendency to positively reframe negative situations. Other authors [3] tested a causal model where gratitude seemed to directly foster social support and protect people from stress and depression. Gratitude has also been positively associated with personality traits such as agreeableness, responsibility, and extraversion, and negatively associated with neuroticism [1, 4].

In adolescents, gratitude has been associated with prosocial behaviour, social integration, and life satisfaction [5]. There were also several studies that support the link between gratitude and satisfactory social relationships [6–8]. Additionally, other studies suggested that gratitude correlated with positive emotions like vitality, subjective happiness, hope, and optimism, and also with well-being and life satisfaction, both in adults and adolescents [1, 9–12]. However, to date no studies have explored the relationship between prototypic symptoms of eating disorders and gratitude in adolescents. Assessing this relationship is relevant because eating disorders constitute a relevant risk factor for developing serious health problems and psychopathology in adulthood and their onset usually occurs during adolescence [13, 14]. Therefore, in terms of promoting mental health it is important to explore whether positive emotions such as gratitude are negatively associated with eating disorders.

Gratitude-based interventions constitute another important research area in this field, serving two main purposes. First, to raise awareness about gratitude as a key component in the promotion of wellbeing in both adults and adolescents [12, 15]. Second, to assess the extent to which gratitude-based interventions could enhance gratitude levels in different populations [10], which would suggest that gratitude is a dispositional quality that may be cultivated and developed [3–8].

Several instruments have been designed to measure gratitude, including: The Gratitude Resentment and Appreciation scale (GRAT; 44 items; [15], and its abbreviated version (16 items; [16]; The Gratitude Adjective Checklist (GAC); [1] which is used to measure gratitude as an emotion, mood, or disposition; The Gratitude Questionnaire-20 items (G20); [17]; and The Gratitude Scale [4], which consists of 18 items that express favourable, neutral, and unfavourable affirmations toward gratitude. However, the most widely used questionnaire which has been validated in several countries, is the Gratitude Questionnaire (GQ- 6) [1], comprising six items.

The GQ-6 is a self-report questionnaire designed to assess individual differences in people’s disposition to experience gratitude in everyday life. Some authors [1] considered gratitude as an affective trait they named grateful disposition. The authors initially developed 39 items (including positive and negative ones) with statements about experiences and expressions of gratitude and appreciation in daily life, among others. Through a series of exploratory and confirmatory factor analyses they developed a robust single factor scale and retained only 6 items that scored high on the first factor, each of them measuring a unique aspect of the grateful disposition. The studies carried out for developing the instrument were conducted with adult and young populations (older than 18), presenting adequate construct validity and reliability [1, 18].

In terms of studies with younger populations (university students and adolescents), one of the most significant adaptations was removing item 6 (“Long amounts of time can go by before I feel grateful to something or someone”) which showed low correlations with the instrument as a whole. For example, in the Taiwan [9] and Turkey [19] GQ-6 validations a 5-item model was found to have a better fit compared to the original 6-item model. Both adaptations showed satisfactory reliability with Cronbach's alphas of .80 and .77, respectively. Froh et al. [20] confirmed these findings in a study that examined whether the GQ-6 (along with the GRAT and GAC) were valid in a sample of adolescents (ages between 10 and 19). The authors reported that the scale presented acceptable internal consistency with alphas higher than .70, and positive correlations between the GQ-6, the GAC and GRAT for all ages, with weaker correlations at younger ages (10 to 13). Regarding Spanish-speaking countries, a preliminary validation of the instrument was carried out with 369 Chilean university students [21]. The results showed a single factor solution with the six items and an adequate reliability (Cronbach’s alpha = 0.74).

To date, the Chilean validation of GQ-6 has been carried out only with a relatively small sample of young people in Chile, but without confirmatory analysis to make the measure more robust. It is still unknown how the instrument behaves in adolescent populations and in adults in Chile. To address this gap and to test the generalizability of previous findings in gratitude research, cross-cultural and international samples are needed. Thus, the purpose of this study is to validate the GQ-6 in both adult and adolescent populations in Chile. To accomplish this, two studies were conducted: Study 1 with high school adolescents, and Study 2 with adults from the general population.

## Methods of study 1

### Participants

The sample consisted of 668 high school students of subsidised-private and paid-private educational institutions of the Metropolitan Region of Santiago, Chile. Schools were selected by convenience sampling and participants from a volunteer sample. Because 3 students dropped out of the study, the final sample consisted of 665 students.

Three hundred and ninety participants were women and 278 were men, with ages ranging between 12 and 20, and a mean age of 15.54 years (*SD* = 1.22). While 35 % of the participants were in the first year of middle school, 29.6 % were in the second year, 23.7 % in the third year, and 11.7 % in the fourth year.

### Questionnaires

#### *The Gratitude Questionnaire* (*GQ-6)*

The GQ-6 [1] is a self-report 6-item scale which assesses individual differences in the tendency to experience gratitude in daily life. Responses range from 1 to 7 on a 7-point Likert-type scale (1 = strongly disagree and 7 = strongly agree). Internal consistency of the instrument ranged between alphas of .76 and .87 [1, 18] (see Additional file 1).

#### The Beck Depression Inventory (BDI-I)

The BDI-I [22] is a self-report 21-item scale which assesses the presence and severity of depressive symptoms. The items are geared towards detecting the cognitive rather than the somatic-vegetative component of depression. Each question has 4 possible answers, assessing the severity and intensity of each symptom, and the total ranges from 0 to 63. Its psychometric properties have been studied exhaustively, showing good internal consistency. In the present study, internal consistency was good, with Cronbach’s alpha of .88.

#### The Eating Attitude Test (EAT-26)

The EAT-26 [23] is an abbreviated version of the EAT-40, a self-report scale which aims to identify typical symptoms and concerns related to eating disorders in non-clinical populations. This test has been used as a screening device to determine eating disorders risk in schools, colleges, and other special risk groups, becoming one of the most widely used standardised tests in the field of eating disorders, with high levels of reliability and validity. The scale presented good psychometric properties in a Chilean simple [24] and a Cronbach’s alpha of .88 was found in the current sample.

### Procedure

The current validation of the Chilean version of the GQ-6 is part of a larger project called “Early detection and treatment of emotional problems in Chilean adolescents and young adults”. The original English version of the GQ-6 was translated from English into Spanish and then back to English using the guidelines proposed by Muñiz and Hambleton [25] for a back-translation method.

This study was approved by the School of Psychology Ethics Committee of the Pontifical Catholic University of Chile. Students were invited to participate voluntarily in this research, and a signed informed consent from them and their parents or tutors were required to participate. Computer-based questionnaires were collectively applied to groups of no more than 20 students during the school day in the computer laboratories of each educational institution. Each application took approximately 35 minutes and was supervised by a researcher who responded to questions and comments from students. The GQ-6, BDI-I and EAT-26 questionnaires were digitised using a web application specifically designed for a larger project in Chile (For details please see: www.myschool4web.cl).

### Data analysis

An analysis of the psychometric properties of the GQ-6 scale was performed to determine the validity and reliability of the instrument in Chilean adolescents and adults. An Exploratory Factor Analysis (EFA) using maximum-likelihood was applied on the items of the GQ-6. Similarly to the procedure followed in the Dutch validation of GQ-6, parallel analyses with Monte Carlo simulations were conducted to determine the number of factors to retain in the EFA [26]. Confirmatory factor analysis (CFA) for the single-factor model of the GQ-6— multivariate normality test was verified through Mardia’s coefficient. In addition, an analysis of invariance by gender was conducted. Cronbach’s alpha coefficient was used as a measure of reliability. Regression and correlation analyses were performed to test for convergent and divergent validity. Statistical analyses were performed using SPSS v. 21 and AMOS v. 21.

## Results

### Construct validity

First, an EFA using Principal Components with the 6 items that comprise the Gratitude Questionnaire was conducted. The results showed two components with eigenvalues greater than 1, which explained 41.13 % and 21.40 % of the variance of the total score, respectively. Given that the item 6 presented the lowest correlation with the other items and that the reliability of the instrument improves without the item, item 6 was removed for the subsequent analysis. After this, a new EFA was performed with 5 items. Data yielded a Bartlett’s coefficient of 814.56, with a *p* < 0.001 and a KMO of .765, confirming the fair use of factor analysis. Anti-image correlation values for individual items were all ≥ 0.68, which is well above the acceptable limit of 0.50. The communalities of the items were adequate, except for item 3. However, because the instrument comprised only a few items, lower communalities can be accepted [27–29]. The analysis extracted a single factor that explained 50.12 % of the variance of the instrument. The results of saturation weights are shown in Table 1. Parallel analysis with Monte Carlo simulations showed a single-factor solution with 5 items (see Table 2).

**Table 1:** Exploratory factor analysis of the one-factor solution by principal components and with varimax rotation

Item	Component 1
1. I have so much in life to be thankful for.	.838
2. If I had to list everything that I felt grateful for, it would be a very long list.	.812
3. When I look at the world, I don’t see much to be grateful for.	.303
4. I am grateful to a wide variety of people.	.701
5. As I get older I find myself better able to appreciate the people, events, and situations that have been part of my life history.	.749
6. Long amounts of time can go by before I feel grateful to something or someone.	.079 (removed)

Items 3 and 6 are reversed

**Table 2:** Monte Carlo parallel analyses from the items of the GQ-6 in adolescents (study 1) and adults (study 2)

Measures	Factor	Raw data	95th percentile	Variance explained (%)
GQ-5 Adolescents	Factor 1	2.551398	1.110693	50.12
	Factor 2	0.939809	1.047406	
GQ-6 Adults	Factor 1	3.550826	1.262570	59.18
	Factor 2	0.781836	1.150883	

After the exploratory analysis, a CFA was conducted. Given that the Mardia coefficient for the CFA was low (19.041), the maximum likelihood method was used to analyse the correlation matrix. To accept or reject the models tested, a combination of goodness of fit indexes were utilised: *χ*^2^/*df,* CFI (*Comparative Fit Index*), TLI (*Tucker Lewis Index*), IFI (*Incremental Fit Index*), RMSEA (*Root Mean Square Error of Approximation*), plus its confidence interval at 90 %, and SRMR (*Standardised Root Mean Square Residual*). Given that *χ*^2^ is very sensitive to sample size [30], *χ*^2^/*df* was used. A quotient of 4 is considered a reasonable adjustment, while values close to 2 are considered as very good [31].

A first CFA tested the structure of the single-factor model represented by six items, revealing adequate goodness of fit indexes, except for *χ*^2^/*df* which was an excessively high value. Given the sample size, other goodness of fit indexes were calculated and delivered acceptable values, except for RMSA, which was too high (see Table 3). Furthermore, regression weights were very low for item 6 (.021) also presenting an excessively high error (.986). In this model, standardised regression weights were statistically significant (*p* < .001), except for item 6 (*p* = .618). These results confirmed that this model was not adequate and that item 6 needed to be removed.

**Table 3:** Goodness of fit indexes for model 1 (6 items) and model 2 (5 items)

Model	*χ* ^2^	*df*	*χ* ^2^/*df*	*p*	n	CFI	TLI	IFI	RMSEA (CI 90 %)	SRMR
Model 1	78.864	9	8.76	.000	665	.919	.865	.919	.108 (.087–.131)	.065
Model 2	21.807	5	4.36	.001	665	.979	.958	.979	.071 (.042–.103)	.029

**Fig. 1:**
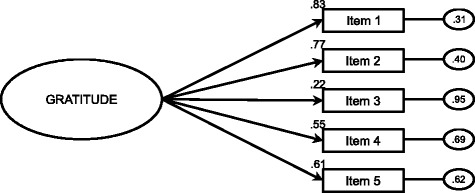
Confirmatory Factor Analysis of the one-factor model with 5 items. Residual variances are shown in the small circles on the right. Note* The oval represents the unique factor and the rectangles represent the five different items. The residual variances are shown in the small circles on the right

After item 6 was removed, a second CFA was carried out with a 5-item model, obtaining a better fit (see Table 3). The results of the goodness of fit indexes confirmed that this model best fits the data (see Fig. 1). It can be noted that incremental indexes (CFI, TLI and IFI) showed a good fit with values of .90 or higher, while error indexes are considered acceptable with values lower than .08 for RMSEA and SRMR, or close to 0.05 to obtain a good fit [32]. Therefore, this one-factor model with 5 items showed a better fit in the current sample than the original one-factor model with 6 items. Consequently, we named this new instrument GQ-5.

In addition we analyzed the invariance of the factor structure as a function of participants’ gender, using multigroup analysis. The results confirmed that the one factor solution of GQ-5 is invariant across the gender of the participants (see Table 4).

**Table 4:** Analysis of invariance by gender (GQ-5)

Models	*χ* ^2^	*gl*	*χ* ^2^/*gl*	Δχ^2^	Δ*gl*	CFI	TLI	IFI	RMSEA	SRMR
Model 1	28.948	10	2.89	-	-	.977	.954	.977	.053	.022
Model 2	43.867	14	3.13	14.91**	4	.964	.948	.964	.057	.050
Model 3	46.753	15	3.11	17.80**	5	.961	.949	.962	.057	.059
Model 4	60.582	20	3.02	31.63***	10	.951	.951	.951	.055	.060

**p < .01; ***p < .001; Model 1: Unconstrained Model; Model 2: Measurement Weights Model; Model 3: Structural Covariances Model; Model 4: Measurement Residuals Model

### Reliability

Cronbach’s alpha for the Chilean version of 5 items was .726, showing an adequate internal consistency for the instrument [33].

### Divergent validity

The relationship between GQ-5 items and the BDI-I (depressive symptoms) and EAT-26 (risk of eating disorder) scores was examined using Pearson’s correlation coefficient for the total sample of adolescents. GQ-5 scores were negatively correlated with BDI-I scores (r = − .345; *p* < .01) and with EAT-26 scores (r = − .106; *p* < .01).

To further explore divergent validity, multiple regression analyses were performed using the total scores of the BDI-I and the EAT-26 as predictors of gratitude score in adolescents. Depressive symptomatology (measured by BDI-I) was weakly associated with the prediction of gratitude in adolescents, explaining only 14 % of the variance. Meanwhile the prototypic symptoms and difficulties of eating disorders (measured by EAT-26) were not associated with gratitude accounting for less than 1 % of the variance, as shown in Table 5.

**Table 5:** Correlations and regression between gratitude in adolescents and mental health outcomes

BDI depressive symptoms			EAT-26 risk of eating disorder
GQ-5	*R* ^*2*^	.141	.011
	*B*	-.228	-.051
	*SE*	.022	.019
	β	-.375	-.106
	*P*	.000	.007

*SE*: Standard error; *β:* Beta standardised

### Methods of study 2

#### Participants

The sample consisted of 331 Chilean adults, of which 231 were women, with an average age of 37.59 years (SD = 12.6). Participants were selected by convenience sampling.

### Questionnaires

#### Gratitude questionnaire (QG-6)

See the description in study 1.

#### Positive and Negative Affect Schedule (PANAS)

PANAS [34] is a 20-item scale, 10 items assessing positive affect, and 10 negative affect. The items consist of words that describe different feelings and emotions. The first Spanish validation was conducted by Sandin et al. [35]. The most recent work and the one used here was developed by De la Rubia [36] in a Mexican population.

#### Depression, Anxiety and Stress Scale (DASS-21)

The DASS-21 [37] has 21 items answered on a 4-point Likert scale (0 = Does not apply to me at all, to 3 = Applies to me very much or most of the time). It is divided into three subscales (with seven items each): depression, anxiety and stress. We used the Chilean validation published by Antúnez and Vinet [38]. The results showed that levels of internal consistency for the subscales of the DASS-21 were .85 for the depression subscale, .73 for anxiety subscale, .83 for stress, and for the total score of the DASS-21 a Cronbach’s alpha of .91.

#### Subjective Happiness Scale (SHS)

The SHS [39] evaluates self-perceived happiness. This scale consists of four items utilising a 7-point Likert scale. In the initial Chilean validation [40], the subjective happiness scale showed acceptable to good internal consistency with Cronbach’s alphas of .73 in adults and .76 in college students. Test-retest reliability showed a relatively low correlation of .61, which might have been influenced by the relatively long gap between both measures (8 weeks). Factor analysis and Varimax rotation for each sample obtained a unique factor, consistent with the English version of this test.

### Procedure

The validation of the scale for gratitude is part of a larger project entitled: ‘Investigating compassion, gratitude and happiness’. This study is a collaborative project developed between different researchers involved in *RedMindfulness*, an online network where Spanish-speaking people interested in the practice of mindfulness interact.

All members of the network (about 2000 people) were invited to participate by email. People who were interested were invited to answer an online questionnaire, which was accompanied by a letter of informed consent. In two months 513 people responded, corresponding to 16 different countries; 331 people were Chileans, which corresponded to 64.5 % of the total sample. This study was approved by the Ethics Committee of the Adolfo Ibáñez University.

### Results

#### Data analysis

See the description of data analysis in Study 1.

### Construct validity

As in Study 1, an EFA using Principal Components with the GQ-6 was conducted. Data yielded a Bartlett’s coefficient of 953.846, with a *p*-value less than 0.001 and a KMO of .854, confirming the appropriate use of factor analysis. Anti-image correlation values for individual items were all ≥ 0.74, which is well above the acceptable limit of 0.50. The communalities of the items were adequate (>0.3). The analysis extracted a single factor that explained 59.18 % of the variance in the data. Parallel analysis with Monte Carlo simulations showed a single-factor solution with 6 items (see Table 2).

**Fig. 2:**
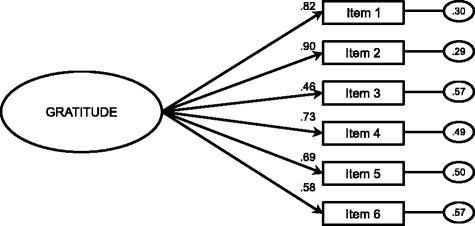
Confirmatory factor analysis GQ-6 in the adult sample. Note* The oval represents the unique factor and the rectangles represent the six different items. The residual variances are shown in the small circles on the right

A CFA was performed to test the model obtained in the EFA. Given that the Mardia coefficient for the CFA was low (54.756), we used a maximum-likelihood estimate method to analyse the correlation matrix. The CFA tested the one-factor model with six items, showing the following adjustment indexes: *χ*^2^ (9, *N* = 331) = 18.195, *p* = .033; *χ*^2^/*df* = 2.02; CFI = .98; TLI = .98; IFI = .98; RMSEA = .056 (IC 90 % = .015-.093); SRMR = .024. In this model, standardised regression weights were statistically significant (*p* < .001), ranging from .46 to .90. The results for different adjustment indexes that were used confirmed that the tested model (i.e., one factor and six items) was the one that best fit the data. Given the CFA characteristics (described in Study 1), a *χ*^2^/*df* coefficient value close to 2 indicated this model presented a good fit (see Fig. 2).

In addition we analyzed the invariance of the factor structure as a function of participants’ gender, using multigroup analysis. The results confirm that the one factor solution of GQ-6 is invariant across the gender of the participants (see Table 6).

**Table 6:** Analysis of invariance by gender (GQ-6)

Models	*χ* ^2^	*gl*	*χ* ^2^/*gl*	Δχ^2^	Δ*gl*	CFI	TLI	IFI	RMSEA	SRMR
Model 1	47.732	18	2.65	-	-	.964	.939	.939	.071	.030
Model 2	58.452	23	2.54	10.72*	5	.957	.943	.943	.068	.068
Model 3	69.119	24	2.88	21.38**	6	.945	.931	.931	.076	.078
Model 4	144.642	30	4.82	96.90***	12	.859	.859	.859	.108	.092

*p < .05; **p < .01; ***p < .001; Model 1: Unconstrained Model; Model 2: Measurement Weights Model; Model 3: Structural Covariances Model; Model 4: Measurement Residuals Model

### Reliability

Cronbach’s alpha was .832, showing a good internal consistency for the instrument [30].

### Convergent and divergent validity

The relationship between GQ-6 with the DASS-21 (depressive, anxiety and stress symptoms), the PANAS (positive and negative affect) and the SHS (Subjective Happiness Scale) scores were examined using Pearson’s correlation coefficient for the total sample of adults. GQ-6 scores were significantly correlated with the three dimensions of the DASS-21, the PANAS, and also with the SHS (see Table 7).

**Table 7:** Correlations and regression between gratitude in adult and mental health outcomes

		DASS Depression	DASS Anxiety	DASS Stress	PANAS Negative affect	PANAS Positive affect	SHS Happiness
*R2* (Total DASS-21) = .303					*R2* (Total PANAS) = .208		*R2* (Total SHS) = .429
GQ-6	*R*	-.529**	-.339**	-.248**	-.331**	.384**	.655**
	*R* ^*2*^	.279	.115	.061	.109	.147	.429
	*B*	-.864	-.233	-.359	−2.110	2.843	.751
	*SE*	.092	.114	.077	.422	.446	.048
	β	-.591	-.134	-.248	-.253	.323	.655
	*P*	.000	.041	.000	.000	.000	.000

*DASS:* depression, anxiety and stress scale; *PANAS:* positive and negative affect schedule; *SHS:* subjective happiness scale; *R: * Pearson correlation; *SE:* standard error; *β: * beta standardised; **p < .01

To further explore the convergent and divergent validity of the test, multiple regression analyses were performed using the DASS-21, the PANAS, and the SHS as predictor variables, and the GQ-6 scale as outcome (see Table 7). The results according to each of the scales are as follows:

1. The factors of DASS-21 scale show that depression, anxiety, and stress are negatively associated with gratitude explaining the 30 % of the variance.
2. The factors of PANAS scale show that positive and negative affect are associated (positively with positive affect and negatively with negative affect) with gratitude explaining the 21 % of the variance.
3. Subjective Happiness Scale: regression analysis showed that subjective happiness factor predicts or is associated positively with gratitude in adults explaining the 43 % of the variance.

## Discussion

The goal of these studies was to validate the Chilean version of the Gratitude Questionnaire (GQ-6) in a sample of Chilean adolescents and adults. As far as we know, this is the first study to present the psychometric proprieties of the GQ-6 for a sample of non-English speaking adolescents and adults of the general population.

On regard to adolescents, in terms of construct validity, the results showed (through CFA) that the best factorial model was one-factor with five items. This solution was consistent with previous GQ-6 validations with adolescents from the USA [20] university/university students from Taiwan and Turkey [9, 19]. The fit of the five-item version of GQ was also confirmed by qualitative findings: adolescents showed difficulties understanding the meaning of item six. More specifically, it seems that the wording of this item might not be fully apprehended by younger participants [20]. In addition, the GQ-5 presented a similar reliability to those reported in the studies mentioned above, with Cronbach’s alphas between .70 and .80.

Study 1 also found appropriate divergent validity of the GQ in relation to psychopathological measures, such as depression (assessed with the BDI-I). These results confirmed that gratitude, as a positive emotion, is negatively associated with depressive symptomatology in adolescents [20, 41]. However, the correlation between gratitude and eating disorders was very weak.

As mentioned above, our rationale to add the EAT-26 scale was that behaviours related to eating disorders constitute relevant risk factors for developing serious health problems and psychopathology in adulthood, and its onset usually occurs during adolescence [13, 14]. But our analysis did not find a relationship between eating disorders (measured by EAT-26) and gratitude. The hypothesis that gratitude may act as a buffer and protective factor for eating disorders should be further studied.

In Study 2 with adults, we found more robust results, with higher reliability and a higher percentage of the variance explained by the model [1, 18]. In other words, the results suggested that the younger the participants, the less reliable and lower capacity of the instrument to assess the disposition of gratitude [9, 19, 20]. Nevertheless, correlations between gratitude and other variables (e.g., optimism, happiness, well-being, spirituality or personality traits) were essentially the same in adults and in university students [1], but in adolescents, these correlations were relatively lower (e.g., between gratitude and positive and negative affect, and life satisfaction [20]. These outcomes were confirmed in the present study. For instance, in Study 2, a positive moderate to strong correlation was found between gratitude and happiness (as measured by SHS), but lower negative correlations were found with the risk of eating disorders for adolescents (Study 1).

### Study limitations and strengths

The limitations of these studies include the fact that, in contrast to the adult sample study, it was not possible to assess the convergent validity of the GQ-5 in the adolescent sample. Therefore, future studies will have to explore the question if gratitude is positively related to happiness, optimism, life satisfaction, positive affect, among other relevant constructs [9, 19]. Also, the five item version of the GQ was found in others’ validations and confirmed by CFA in this study, but this version was not reapplied in an adolescent sample to test its psychometric properties as recommended [42]. The size of the adult sample was relatively small and unbalanced in terms of participants’ gender. Our analysis might have also been enhanced by using additional questionnaires that measured gratitude. Unfortunately, no other validated gratitude scales are available in Chile, which is precisely one of the identified gaps this article tried to address.

The relevance of research on gratitude is supported by recent evidence that suggests that regular experiences of positive emotions can make people more resilient and healthier, reinforcing an upward spiral of optimum performance [43]. We believe that gratitude can be enhanced and trained to promote and reinforce this kind of upward spiral in people’s lives. Gratitude could serve as a powerful psychological buffer to enhance resilience and well-being [1, 10, 11, 44], particularly in young people facing relevant risk factors for mental health, including depression and addictions [12, 45].

The validation of the Chilean version of the GQ-6 will facilitate cross-cultural and international comparison of gratitude research outcomes, promoting a better understanding of the cultural similarities and differences in the way this concept is construed in different cultures. Additionally, providing a reliable measure for the assessment of gratitude in Chilean adolescents and adults will allow researchers, healthcare professionals, educators, and policy makers, to develop and measure the effects of interventions aimed at improving the levels of wellbeing and life quality in a country with one of the highest levels of mental health problems in the OECD [46].

## Conclusions

The Chilean adaptations of the GQ (GQ-5 and GQ-6) showed good psychometric properties, similar to those of the original version and the previous validations in other countries and cultural contexts. Therefore, the GQ is a suitable measure to evaluate a person’s disposition toward gratitude in Chilean adolescents, young adults [19] and adults. This instrument may contribute to the study of positive emotions and human development in Latin America.

## Additional file

Additional file 1: Chilean translation of the Gratitude Questionnaire (GQ-6). (DOCX 14 kb)
