# Supplementary material for: Validation of a Spanish translation of the Gratitude Questionnaire (GQ-6) with a Chilean sample of adults and high schoolers
Source: Health Qual Life Outcomes. 2016 Mar 31;14:53. doi: 10.1186/s12955-016-0450-6 (PMC4815209; doi:10.1186/s12955-016-0450-6)
Supplement: Additional file 1: — Chilean translation of the Gratitude Questionnaire (GQ-6). (DOCX 14 kb) [file 12955_2016_450_MOESM1_ESM.docx]

**Supplementary Material**

Validation of a Spanish translation of the Gratitude Questionnaire (GQ-6) with a Chilean sample of adults and high schoolers

| **Chilean translation of GQ-6** |
| --- |
| 1. Tengo muchas cosas en mi vida por las cuales estar agradecido/a. |
| 2. Si tuviera que hacer una lista de todo lo que me hace sentir agradecido/a, sería una lista larga. |
| 3. Cuando observo el mundo, no veo mucho por lo que estar agradecido/a. |
| 4. Estoy agradecido/a de una amplia variedad de personas. |
| 5. A medida que me hago mayor, me encuentro más capaz de apreciar las personas, los eventos y las situaciones que han sido partes de mi vida. |
| *6. Puede pasar una gran cantidad de tiempo antes de que me sienta agradecido/a por algo o alguien. |

Note: *in a sample of adolescents the item 6 needs to be removed
